# Quantification of mitral annular excursion from cine MRI and validated by Doppler echocardiography

**DOI:** 10.1186/1532-429X-16-S1-P20

**Published:** 2014-01-16

**Authors:** Shahryar G Saba, Vandana Sachdev, Hwaida Hannoush, Leon Axel, Andrew E Arai

**Affiliations:** 1 National Heart, Lung, and Blood Institute, Bethesda, Maryland, USA; 2 Department of Radiology, New York University Langone Medical Center, New York, New York, USA

## Background

Mitral annular plane systolic excursion (MAPSE) reflects longitudinal left ventricular function, predicts survival in heart failure patients and provides a sensitive marker of early systolic dysfunction in hypertensive patients with normal ejection fraction (EF). Doppler mitral annulus velocity (e') in early diastole predicts cardiac mortality, differentiates diseases and estimates filling pressures. We present a practical technique that facilitates measuring atrioventricular junction (AVJ) motion throughout the cardiac cycle from cine cardiac magnetic resonance (CMR) and correlate these measurements with EF and echocardiography-determined e'.

## Methods

**Figure 1:**
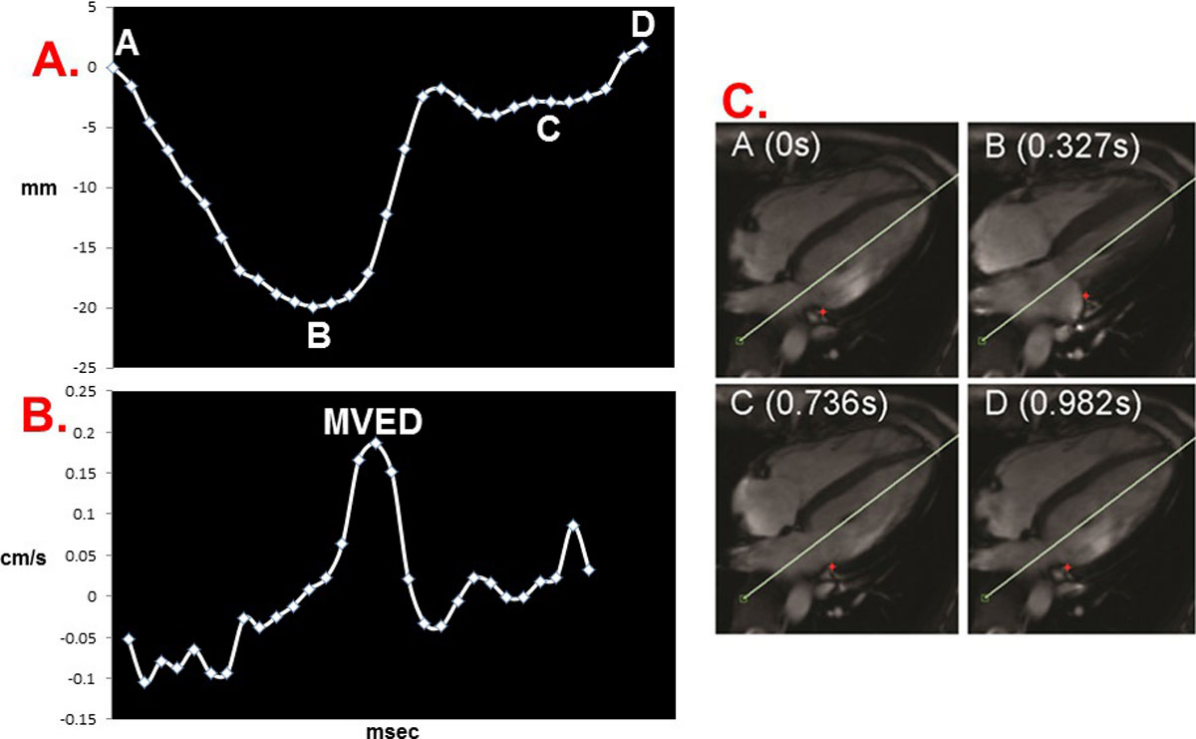
**AVJ Displacement-Versus-Time (A) and Velocity-Versus-Time (B) Plot and Images (C)**. A. Atrioventricular junction motion tracking software measures the longitudinal AVJ position at multiple time points during the cardiac cycle. Lateral AVJ position, marked with a red asterisk, shown at the start of systole (0 s), end systole (0.327 s), diastasis, (0.736 s) and end diastole (0.982 s) for a representative 4-chamber cine image set. AVJ = atrioventricular junction; MVED = maximum velocity early diastole

**Figure 2:**
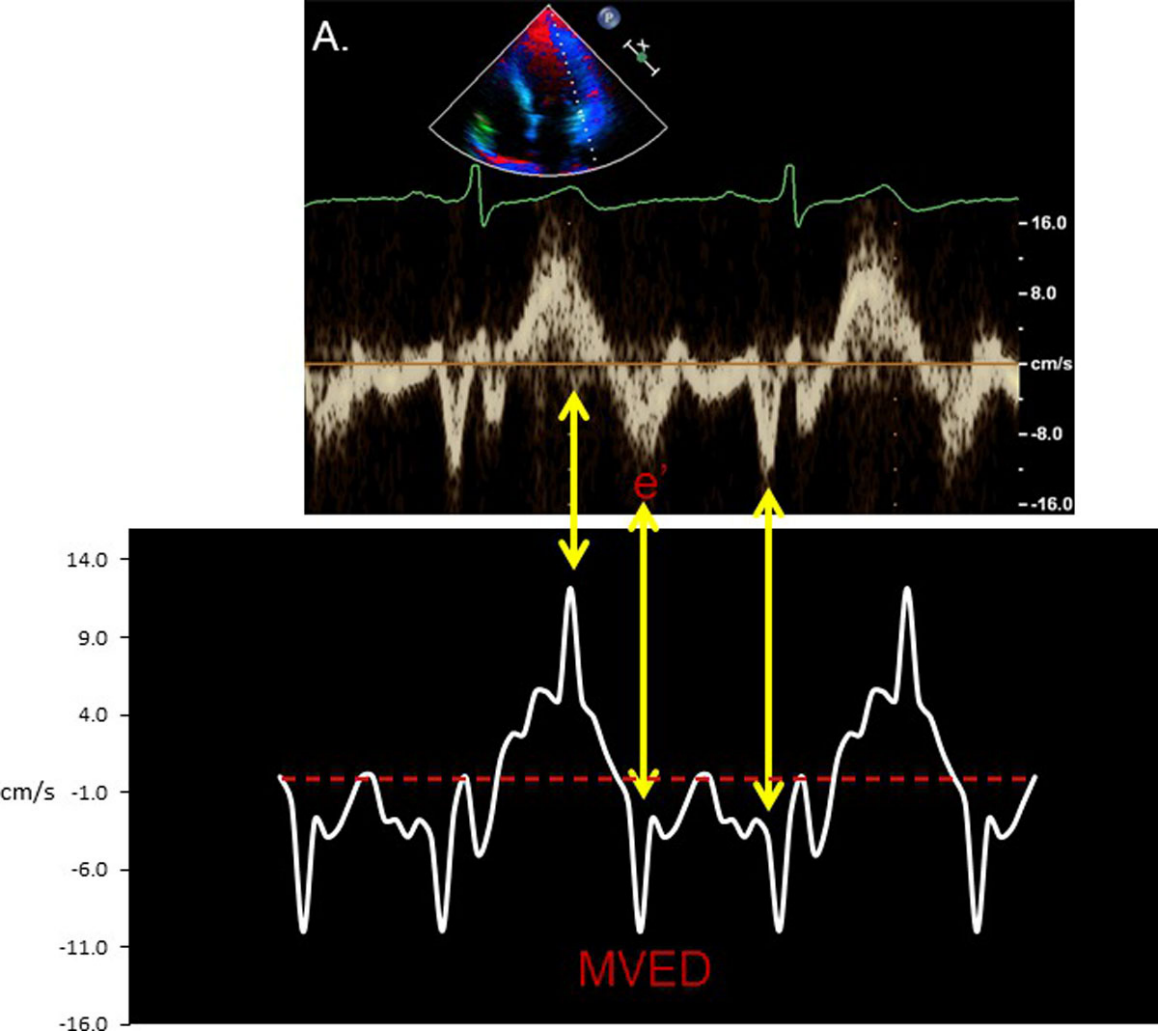
**TDE Mitral Annulus and CMR AVJ Velocity Correlation**. A. TDE demonstrates s', e' and a' waves corresponding to mitral annular velocities during systole, early diastole and with atrial contraction, respectively for a representative patient. B. Corresponding CMR-derived AVJ velocity-versus-time curve. Bidirectional arrows demonstrate corresponding mitral annular velocities during various phases of the cardiac cycle. AVJ = atrioventricular junction; MVED = mitral velocity early diastole; TDE = tissue Doppler echocardiography.

Twenty-seven patients underwent CMR and transthoracic echocardiography (TTE) within 24 hours, between June 2010 and September 2013. Using custom-written MATLAB software we tracked AVJ motion throughout the cardiac cycle, using 4-chamber cine images (Figure 1). The longitudinal AVJ displacement was plotted as a function of time, and maximum systolic displacement and early diastolic velocity were determined (Figures 1 and 2). Three separate tissue Doppler echocardiography (TDE) measurements of lateral e' were averaged. Pearson's correlations between the maximum longitudinal displacement (MD) of the AVJ and CMR-derived EF, as well as the maximum velocity of the AVJ in early diastole (MVED) and e' were calculated.

## Results

Seventeen woman and 10 men with a mean age of 59 (range 36-82) underwent CMR and TTE. Mean values for CMR-derived EF and TDE e' were found to be 53% (range 28-71% and 11 cm/s (range 3-18 cm/s), respectively. Moderate correlations were found between MVED and e' (r = 0.624, p = 0.001) as well as EF and MD (r = 0.486, p = 0.01). A moderate correlation was also found for MD and left ventricular stroke volume (r = 0.504, p = 0.007). Atrioventricular junction analysis was performed offline and took approximately 10 minutes per patient.

## Conclusions

Atrioventricular junction analysis is a practical CMR technique that yields CMR measurements of diastolic and systolic function. A moderately strong correlation was found between CMR-derived MVED and e', as well as EF and MD.

## Funding

Supported by the National Heart, Lung and Blood Institute, National Institutes of Health by the Division of Intramural Research, NHLBI, NIH, DHHS.

